# Implementation and engagement of the SMART Work & Life sitting reduction intervention: an exploratory analysis on intervention effectiveness

**DOI:** 10.1186/s12966-023-01548-5

**Published:** 2023-12-19

**Authors:** Charlotte L Edwardson, Lucy Abell, Alex Clarke-Cornwell, David W Dunstan, Laura J Gray, Genevieve N Healy, Michelle Hadjiconstantinou, Panna Wilson, Benjamin Maylor, Fehmidah Munir, Stuart JH Biddle

**Affiliations:** 1 Diabetes Research Centre, University of Leicester, Leicester, LE5 4PW UK; 2 NIHR Leicester Biomedical Research Centre, Leicester, LE5 4PW UK; 3 Department of Population Health Sciences, University of Leicester, Leicester, LE1 7RH UK; 4 School of Health & Society, University of Salford, Salford, Greater Manchester, M6 6PU UK; 5 Baker Heart and Diabetes Institute, Melbourne, VIC 3004 Australia; 6 Institute for Physical Activity and Nutrition, School of Exercise and Nutrition Sciences, Deakin University, Melbourne, VIC 3125 Australia; 7 School of Human Movement and Nutrition Sciences, The University of Queensland, Brisbane, QLD 4072 Australia; 8 Leicester Diabetes Centre, University Hospitals of Leicester, Leicester, LE5 4PW UK; 9 School of Sport, Exercise and Health Sciences, Loughborough University, Leicestershire, LE11 3TU UK; 10 Centre for Health Research, University of Southern Queensland, Springfield Central, QLD 4300 Australia; 11 Faculty of Sport & Health Sciences, University of Jyväskylä, Jyväskylä, FI-40014 Finland

**Keywords:** Engagement, Fidelity, Sitting, Intervention, Workplace

## Abstract

**Background:**

To enhance the impact of interventions, it is important to understand how intervention engagement relates to study outcomes. We report on the level of implementation and engagement with the SMART Work & Life (SWAL) programme (delivered with (SWAL plus desk) and without a height-adjustable desk (SWAL)) and explore the effects of different levels of this on change in daily sitting time in comparison to the control group.

**Methods:**

The extent of intervention delivery by workplace champions and the extent of engagement by champions and participants (staff) with each intervention activity was assessed by training attendance logs, workplace champion withdrawal dates, intervention activities logs and questionnaires. These data were used to assess whether a cluster met defined criteria for low, medium, or high implementation and engagement or none of these. Mixed effects linear regression analyses tested whether change in sitting time varied by: (i) the number of intervention activities implemented and engaged with, and (ii) the percentage of implementation and engagement with all intervention strategies.

**Results:**

Workplace champions were recruited for all clusters, with 51/52 (98%) attending training. Overall, 12/27 (44.4%) SWAL and 9/25 (36.0%) SWAL plus desk clusters implemented all main intervention strategies. Across remaining clusters, the level of intervention implementation varied. Those in the SWAL (n = 8 (29.6%) clusters, 80 (32.1%) participants) and SWAL plus desk (n = 5 (20.0%) clusters, 41 (17.1%) participants) intervention groups who implemented and engaged with the most intervention strategies and had the highest percentage of cluster implementation and engagement with all intervention strategies sat for 30.9 (95% CI -53.9 to -7.9, *p* = 0.01) and 75.6 (95% CI -103.6 to -47.7, *p* < 0.001) fewer minutes/day respectively compared to the control group at 12 month follow up. These differences were larger than the complete case analysis. The differences in sitting time observed for the medium and low levels were similar to the complete case analysis.

**Conclusions:**

Most intervention strategies were delivered to some extent across the clusters although there was large variation. Superior effects for sitting reduction were seen for those intervention groups who implemented and engaged with the most intervention components and had the highest level of cluster implementation and engagement.

**Trial Registration:**

ISRCTN11618007. Registered on 24 January 2018. https://www.isrctn.com/ISRCTNISRCTN11618007.

**Supplementary Information:**

The online version contains supplementary material available at 10.1186/s12966-023-01548-5.

## Background

It has been two decades since the UK’s Medical Research Council (MRC) first published its framework for complex interventions [1], with revised and updated guidance issued in 2008 [2] and 2021 [3]. One important feature of this guidance is the recognition of the implementation of an intervention and the use of process evaluation to determine why an intervention may have worked well or not. Moreover, the MRC has also produced guidance for the development and conduct of process evaluations [4]. This guidance highlights the importance of understanding both the delivery (implementation) of the intervention and the potential mechanisms of impact. Typically, focus of process evaluations has been on understanding delivery aspects, such as intervention fidelity. Less is reported on the engagement of the participants in elements of the intervention that are delivered, despite engagement being important for identifying mechanisms of impact [4]. For example, it has previously been shown that a higher number of logins to a programme (an indicator of higher engagement) led to greater increases in physical activity [5–7], however some research has contradicted this [8, 9].

It is not only important to assess the extent of intervention engagement through process evaluation but also understand how this then relates to study outcomes, thus providing an opportunity to enhance the impact of interventions. A recent review found a positive but weak association between engagement with a physical activity digital health intervention and physical activity outcomes, but no studies in the review targeted sedentary behaviour outcomes [10]. Understanding engagement in an intervention that specifically targets reductions in sedentary time is important as the determinants of sedentary behaviour and physical activity have been shown to vary [11]. Moreover, in a common setting for sedentary reduction interventions (i.e., the desk based workplace), the influences on behaviour (and thus the potential influences on engagement) are likely to vary across organisations and work groups. Leonard et al. [12] have reported on explanatory factors that might help explain why some worksites performed better than others in a sedentary time reduction intervention. Here, both intervention adherence and competence of the person implementing the intervention were important in discriminating high from low performing worksites for sedentary time reduction. But no data on engagement were reported.

The SMART Work & Life intervention (SWAL), – the focus of the present paper – delivered with and without a height-adjustable workstation, was designed to reduce sitting time during and outside of work hours in ambulatory desk-based workers. Workplace champions, defined as employees within the organisation delivering the intervention, were trained to facilitate the delivery of SWAL and intervene at the social network level and promote social support [13]. This approach has been shown to be more effective at promoting behaviour change than others delivering interventions in the workplace [14]. A cluster randomised controlled trial demonstrated that SWAL successfully reduced daily sitting time over 3 and 12 months [15]. The experiences of the workplace champions delivering SWAL and participants taking part have been reported previously [16]; these were largely positive but some intervention strategies were perceived as more useful than others.

In SWAL, some BTCs had an element of choice of strategies to use (e.g., what small environmental changes to make). Hence these could vary across clusters. However, the main intervention activities, and when they should be delivered, was set to a defined schedule. The process evaluation highlighted that intervention implementation varied by each workplace champion as well as the extent of participant engagement with each component [15]. It is likely that this variation could have impacted intervention effectiveness. Furthermore, examining the delivery of the intervention, and the interventionists’ fidelity to the protocol on programme outcomes, has been identified as being especially crucial when the intervention is delivered by Champions in ‘real-world’ settings [17]. Understanding how engagement impacted outcomes will also provide insight into whether all components were needed to achieve intervention success. This could be important information which may help minimise the intervention demands and reduce the burden for participants and the delivery itself.

The aims of this paper, therefore, are to report on the level of implementation and engagement with the SWAL intervention and present an exploratory analysis to examine the effects of different levels of intervention implementation and cluster engagement on intervention effectiveness (i.e., change in daily sitting time) in comparison to the control group. Two elements were tested: Whether change in sitting time varied by i). the number of intervention strategies implemented and engaged, and ii). the percentage of implementation and engagement with all intervention strategies.

## Methods

### Design, setting and participants

**Fig. 1:**
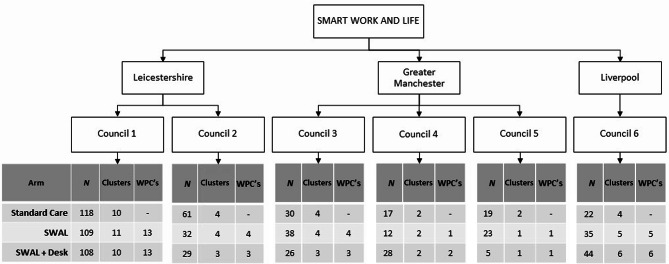
Flow of clusters, participants and workplace champions within each council involved in the study

The main study was a three-arm cluster randomised controlled trial to evaluate the effectiveness of the SWAL sitting reduction intervention, delivered with and without a height-adjustable workstation. The main trial protocol has been described in detail elsewhere [18] along with the main effectiveness [15] and cost effectiveness results [19]. Ethical approval for the trial was received from the University of Leicester’s College of Life Sciences (Ref:14,372). The current analysis utilises data from this randomised controlled trial to explore whether different levels of intervention implementation and engagement impacts the main outcome - change in daily sitting time in comparison to the control group. The target population for the main trial were ambulatory office workers in local government Councils close to the research sites of Leicester and Salford in the UK. Office workers within six local Councils were eligible to take part in the study if they were ≥ 18 years old, spent the majority (≥ 50%) of their day sitting, worked at least 60% full-time equivalent (e.g., ≥ 21 h for those on a 35 h working week contract), were willing to give informed consent, and were able to walk unassisted. Participants were not eligible if they were pregnant, already used a height-adjustable workstation, or were unable to provide informed consent or communicate in English. Office workers who were interested in taking part in the study completed an information form which was used to assess eligibility and provide detail for determining clusters. To be eligible, each cluster was required to have at least one participant who was willing to act as workplace champion. Participants were asked to indicate on the information form if they were willing to take on this role. Appropriate clusters were determined by their desk/office location with multiple clusters allowed per site. Clusters were randomised to either the SMART Work & Life intervention (SWAL only), the SMART Work & Life intervention with the addition of a height-adjustable workstation (SWAL plus desk), or the control group, who continued with usual practice. Figure 1 shows the number of clusters, participants and workplace champions taking part within each council. Data were collected at baseline and at 3 and 12 month follow up. All participants provided informed consent prior to baseline measurements.

### SMART Work & Life intervention

SWAL was grounded in several theories, including Social Cognitive Theory [20], Organisational Development Theory [21], Habit Theory [22], Self-Regulation Theory [23], and Relapse Prevention Theory [24]. It also drew upon the principles of the Behaviour Change Wheel and the associated COM-B approach [25] and the use of different behaviour change techniques (BCTs) [26] shown to be promising for sedentary behaviour reductions and worksite-delivered studies [27]. It has been described in detail within the main results paper [15] but, in brief, the intervention aimed to reduce sitting time during and outside of work hours. The intervention consisted of organisational (e.g., support of senior leaders, workplace champions driving the delivery, monthly emails), environmental (small scale restructuring, posters), group (e.g., group catch up sessions, sitting less challenges), and individual (e.g., online education programme, self-monitoring and prompts) behaviour change strategies. Both intervention arms received the same intervention but the intervention plus height-adjustable desk arm received a height-adjustable workstation of their choice from four models. In SWAL, workplace champions (one or two per cluster depending on the size of cluster) were trained to facilitate the intervention and were provided with training and support (from a team within the Leicester Diabetes Centre (independent of the core research team) experienced in providing training for delivering structured education programmes), intervention resources, and a suggested schedule for delivering each strategy (Table 1). The research team had no involvement in the intervention delivery after the workplace champions were trained.

**Table 1:** Suggested intervention delivery schedule for workplace champions

	Month											
	1	2	3	4	5	6	7	8	9	10	11	12
Intervention launch	X											
Online education for individual staff and manager (with links to self-monitoring tools and prompts)	X											
Monthly emails	X	X	X	X	X	X	X	X	X	X	X	X
Sitting less and moving more challenges	X		X						X			
Group catch up sessions			X						X			
Motivational posters displayed	X	X	X	X	X	X	X	X	X	X	X	X
Height-adjustable workstation with guidance booklet (SWAL + Desk group only		X	X	X	X	X	X	X	X	X	X	X

### Measures

#### Intervention implementation and engagement

The extent to which each workplace champion delivered the intervention activities and the extent to which the participants engaged with each intervention activity was assessed via several methods:

1. attendance logs at the training provided for workplace champions. Each cluster was required to have an individual attend face-to-face training (took place at their workplace) to become a workplace champion for the intervention;.
2. dates of when a workplace champion withdrew from their role and therefore left the cluster without a champion if a replacement could not be found;.
3. dates of delivery of intervention activities (champion). The workplace champions were provided with a set schedule on when to deliver each of the main intervention activities (Table 1 presents a summary of the schedule). Workplace champions were asked to enter dates of when they delivered each intervention activity over the 12 month period and submit this to the research team at three intervals during the study;.
4. all participants were asked to complete a questionnaire at 3 and 12 month follow up concerning their completion of the online education session, use of the height-adjustable workstation (if randomised to that arm), use of the suggested self-monitoring tools and prompts, attendance at group catch-up sessions, and involvement in the sitting less challenges.

The first aim of our exploratory analysis was to explore whether change in sitting time varied by the number of intervention components that were implemented and engaged with. The three levels presented in Table 2 were defined by the study team after database lock, but prior to this analysis. To meet the criteria for the top level (Level 1) of intervention implementation and engagement, a cluster was expected to have implemented and engaged with all of the planned main intervention strategies. Specifically, they must have had a workplace champion trained and for them to remain in the role for ≥ 3 months, the champion must have delivered a high percentage of the ongoing support strategies (i.e., monthly emails, group catch up session and sitting less challenges), and a high percentage of the cluster (≥ 75%) must have engaged with the individual strategies (online education, self-monitoring, and height-adjustable desk use (SWAL plus desk group only)), with the level of engagement required specified in Table 2. The medium level (Level 2) was the same as the top level but the requirement to engage with the self-monitoring strategy was removed. The low level (Level 3) was the same as the medium level but the requirement to implement the ongoing support strategies (emails, group catch up and challenges) was removed. If a cluster did not meet even the lowest level of implementation and engagement (i.e., level 3) they were defined as limited or no implementation and engagement (Level 4).

**Table 2:** Levels summarising the number of intervention components implemented and engaged with

Intervention components	Level of implementation and engagement		
	Level 1 (top level)	Level 2 (medium level)	Level 3 (low level)
Cluster had a workplace champion trained and stayed in the role past the first 3 months	✓	✓	✓
≥ 75% of the cluster completed the online education^a^	✓	✓	✓
Workplace champion sent ≥ 75% of monthly emails OR delivered at least 1 out of 2 group catch ups OR delivered at least 2 out of 3 sitting less challenges (these activities were ongoing support components)	✓	✓	
≥ 75% of the cluster used any self-monitoring tool	✓		
≥ 75% of the cluster reported using the height-adjustable workstation > few times a week (SWAL plus desk group only)	✓	✓	✓

^*a*^ *by 12 month data collection, ≥ 75 of the cluster*

^*b*^ *to have delivered these strategies*

^*c*^ *data collected at the 3 and 12 month follow ups were considered*

^*d*^ *desk use reported at 12 month follow up was used*

The second aim was to explore whether change in sitting time varied by the percentage of cluster intervention implementation and engagement with all planned intervention activities. Again, we defined three levels. At each level all intervention components were held constant but the percentage of implementation by the champion and engagement by participants was changed, as highlighted in Table 3. The top level (Level 1) was kept the same as in Table 2, with the champion implementing a high percentage of the ongoing support strategies (i.e., sending ≥ 75% of the emails) and ≥ 75% of the cluster engaging with the individual strategies. For the medium (Level 2) and low (Level 3) levels, the percentage of cluster implementation and engagement was reduced to ≥ 50% and ≥ 25% respectively. For example, this means that for the medium level the champion must have implemented ≥ 50% of the intervention component, sent ≥ 50% of the monthly emails, and ≥ 50% of the cluster must have completed the online education. If a cluster did not meet even the lowest level (i.e., Level 3) as defined previously they were defined as limited or no implementation and engagement (Level 4).

**Table 3:** Levels summarising the percentage of implementation and engagement with each intervention component

Intervention components	Level of implementation and engagement of each intervention component		
	Level 1 (top level)	Level 2 (medium level)	Level 3 (low level)
Cluster had a workplace champion trained and stayed in the role past the first 3 months	✓	✓	✓
≥X% of the cluster completed the online education	≥ 75%	≥ 50%	≥ 25%
Workplace champion sent ≥ X% of monthly emails OR delivered 1 out of 2 group catch ups OR delivered 2 out of 3 sitting less challenges (these activities were ongoing support components)	≥ 75%	≥ 50%	≥ 25%
≥X% of the cluster used any self-monitoring tool	≥ 75%	≥ 50%	≥ 25%
≥X% reported using the height-adjustable workstation > few times a week (SWAL plus desk group only)	≥ 75%	≥ 50%	≥ 25%

#### Daily sitting time

The activPAL3™ micro accelerometer-based device (PAL Technologies Ltd, Glasgow, Scotland, UK) was used to assess average daily (i.e., all waking hours) sitting time. Proprietary algorithms, within the PAL Technologies software, classify postures (i.e., lying/sitting, upright), transitions between these postures and stepping based on angle of the thigh and static and dynamic acceleration. Participants were asked to wear the device 24 h a day for 8 days whilst also completing a sleep diary which recorded the times they got into bed, went to sleep, woke up and got out of bed. The activPAL was initialised with default settings and the device was waterproofed with a nitrile sleeve and attached to the midline anterior aspect of the thigh using a transparent dressing. On device return, data were downloaded using PAL Connect and event files, using the VANE algorithm, were exported. Event files were then uploaded into the freely available Processing PAL java application (University of Leicester, Leicester, UK available at: https://github.com/UOL-COLS/ProcessingPAL) to be cleaned and processed as described in Edwardson et al. [15]. Once data were cleaned, average daily sitting time was calculated from any valid days. A valid day was defined as having ≥ 10 h wear time per day, ≥ 1,000 steps per day, and < 95% of the day spent in any one behaviour. To be included in the analysis participants were required to have at least one valid day (could be any day of the week).

### Statistical analysis

Here we present the results from a secondary analysis of the intervention arms of a completed randomised controlled trial [15]. This analysis was not part of the original statistical analysis plan for the trial, and a separate prospective analysis plan for the analyses presented here was not written prior to undertaking the work; therefore, these results should be viewed as hypothesis generating.

Basic descriptive statistics were calculated for each cluster on implementation and engagement of each intervention component. These were used to group clusters into each level outlined in Tables 2 and 3.

In the main trial analysis the primary outcome, daily sitting time at 12 months on any valid day (minimum 1 day), was analysed on a complete case basis (i.e., only participants with valid activPAL data were included) using a linear multilevel model. Sitting time at 12-month follow-up was included as the outcome, adjusting for daily sitting time at baseline and average valid activPAL waking wear time across baseline and 12-month follow-up. The model also included a categorical variable for randomisation group (control as reference), and terms for the stratification factors (area: Leicester, Greater Manchester and Liverpool, and cluster size). Office clusters were included as a random effect. The analysis presented here, replicates that undertaken for the main trial results but excludes clusters based on their intervention implementation and engagement.

## Results

For the main trial, 78 clusters with a total of 756 participants were randomised of which 26 (267 participants), 27 (27 champions; 249 participants) and 25 (29 champions; 240 participants) clusters were randomised to the control, SWAL only and SWAL plus desk arms respectively. Participants were on average 44.7 ± 10.5 years of age, 72.4% were female, 74.9% were of white ethnicity, worked 35.4 (3.6) hours per week, had worked at the Council for a 12.4 (9.4) years and shared an office with 59.4 (61.1) people. Information on intervention implementation and engagement was not returned by all workplace champions and participants. Table 4 outlines the data received to assess intervention implementation and cluster engagement. Workplace champion training attendance data were available for all clusters, with 51/52 clusters having at least one champion trained (one cluster from SWAL only did not have a champion trained). Within the first three months of the study, four champions (two from SWAL only and two from SWAL desk) withdrew from the role with no replacement being found (9.6% of clusters without a champion). Of the 51 clusters who had champions trained, 47 clusters returned intervention implementation records for at least one time point throughout the study period. Questionnaires asking about engagement with each main intervention component were received from 84.9% to 87.2% of participants who were seen at 3 and 12 month follow up respectively. In the SWAL plus desk group, implementation records were returned from all workplace champions for at least one time point during the study but not all champions (82%) returned their implementation records in the SWAL only group.

**Table 4:** Available data for assessing intervention implementation and cluster engagement

Type of data	Number of participants and clusters	
	SWAL	SWAL + Desk
Workplace champion training attendance	29 workplace champions from 26 (96%) clusters	27 workplace champions from 25 (100%) clusters
Questionnaire at 3 month follow up on each intervention component	180 participants (78% of those still in the study; 83% of those who attended 3 month follow up) 27 (100%) clusters	191 participants (85% of those still in the study at 3 months; 87% of those who attended 3 month follow up) 25 (100%) clusters
Questionnaire at 12 month follow up on each intervention component	162 participants (73% of those still in the study; 86% of those who attended 12 month follow up) 27 (100%) clusters	178 participants (80% of those still in the study at 3 months; 88% of those who attended 12 month follow up) 25 (100%) clusters
Workplace champion implementation records received at either 3, 9 or 15 months*	22 clusters (82%)	25 clusters (100%)

*These documents were requested at 15 months to understand what had been delivered by the 12-month follow-up

Overall, 12/27 (44.4%) SWAL only and 9/25 (36.0%) SWAL plus desk clusters (i.e., champions) implemented all of the main intervention strategies (sent link to online education, sent all monthly emails, all group catch up sessions (n = 2) and all sitting less challenges (n = 3)). In the SWAL only group, four (14.8%) champions did not implement any of the strategies, this was zero in the SWAL plus desk group. Table 5 summarises the level of implementation and engagement for the two intervention groups. Overall, the online education session was completed by 100% of participants in 17/52 (32.7%) clusters, with 81% of participants in both intervention groups completing this component. All monthly emails were sent by 27/52 (51.9%) clusters. All three sitting less challenges were conducted by 28/52 (53.8%) clusters and both group catch-up sessions were conducted by 31/52 (59.6%) clusters. Only 6 clusters had 100% of participants use a self-monitoring or prompt tool, with approximately a third of participants in both intervention groups reporting using at least one of suggested self-monitoring or prompt tools, this increased when including additional tools participants may have sought themselves. In the SWAL plus desk group, 81.1% of participants within the clusters reporting using their height-adjustable workstation at least a few times per week.

**Table 5:** Implementation of and engagement with the intervention strategies

	SWAL only (n = 27 clusters)		SWAL plus Desk (n = 25 clusters)	
**Education session completion (% of participants)**	80.9%		80.5%	
**Emails sent (number of clusters)**				
100%	14 (51.9%)		13 (52.0%)	
75–99%	5 (18.5%)		4 (16.0%)	
50–74%	3 (11.1%)		1 (4.0%)	
< 50%	5 (18.5%)		7 (28.0%)	
**Group catch up sessions**				
2	16 (59.3%)		15 (60.0%)	
1	6 (22.2%)		6 (24.0%)	
0	5 (18.5%)		4 (16.0%)	
Participants reporting attending	111 (70.7%)		112 (62.9%)	
**Sitting less challenges**				
3	14 (51.9%)		14 (56.0%)	
2	7 (25.9%)		4 (16.0%)	
1	2 (7.4%)		4 (16.0%)	
0	4 (14.8%)		3 (12.0%)	
Participants reporting taking part	58 (38.2%)		50 (28.6%)	
**Self-monitoring tools**	3 months	12 months	3 months	12 months
Suggested	38.6%	31.4%	29.3%	27.7%
Suggested plus additional	62.7%	47.2%	44.7%	45.8%
**Height-adjustable desk use**	Not applicable			
Everyday			80.1%	52.5%
Few times a week			16.0%	30.4%

Tables 6 and 7 summarise the level of implementation and engagement for all clusters and by level of implementation and engagement. Only 13 (25.0%) clusters met the top level (Level 1) of intervention implementation and cluster engagement (Tables 6 and 7), with 42.3% (Table 6) and 17.3% (Table 7) of clusters not even meeting the ≥ low level (Level 3) of intervention implementation and cluster engagement for the number (Table 6) and percentage (Table 7) of intervention components implemented and engaged with respectively. There was still reasonable implementation and engagement with some aspects of the intervention components from many of these clusters when examining the number of intervention components (Level 4, Table 6); however, this was not the case for percentage of implementation and engagement (Level 4, Table 7).

**Table 6:** Level of implementation and engagement determined by number of intervention components (as outlined in Table 2)

	Level 1: High implementation and engagement		Level 2: ≥Medium^a^ implementation and engagement		Level 3: ≥Low^b^ implementation and engagement		Level 4: Limited or no implementation and engagement	
Intervention components	SWAL	SWAL + desk	SWAL	SWAL + desk	SWAL	SWAL + desk	SWAL	SWAL + desk
**Clusters** **Participants**	**8 (29.6%)** **80 (32.1%)**	**5 (20%)** **41 (17.1%)**	**17 (63%)** **163 (65.5%)**	**11 (44%)** **111 (46.3%)**	**17 (63%)** **163 (65.5%)**	**13 (52%)** **142 (59.2%)**	**10 (37%)** **86 (34.5%)**	**12 (48%)** **98 (40.8%)**
No. of clusters who had a workplace champion trained and stayed in the role past the first 3 months	8 (100%)	5 (100%)	17 (100%)	11 (100%)	17 (100%)	13 (100%)	6 (60%)	11 (91.67%)
Average percentage of participants within the clusters who completed the online education	91.81% (range 77.78–100%)	94.44 (range 83.33–100%)	92.65% (range 77.78–100%	92.88% (range 80.0-100%	92.65% (range 77.78–100%	90.34% (range 75.0-100%)	50.08% (range 0-71.43%)	68.18% (range 0-100%)
Average percentage of the monthly emails sent by workplace champions	81.0% (range 30.0-100%	88.4% (range 60.0-100%)	86.5% (range 30.0-100%)	88.8% (range 44.4–100%)	86.5% (range 30.0-100%)	80.6% (range 30.0-100%)	52.3% (range 0-100%)	71.1% (range 0-100%)
No. of clusters who delivered 1 out of 2 group catch ups	8 (100%)	5 (100%)	16 (94.12%)	11 (100%)	16 (94.12%)	11 (84.6%)	6 (60%)	10 (83.3%)
No. of clusters who delivered 2 out of 3 sitting less challenges	7 (87.5%)	5 (100%)	16 (94.12%)	10 (90.91%)	16 (94.12%)	10 (76.92)	5 (50%)	8 (66.67%)
Average percentage of the cluster who used any self-monitoring tool	86.7% (range 75.5–100%)	85.1% (range 75.0-100%)	68.7% (range 0-100%)	66.1% (range 16.7–100%)	68.7% (range 0-100%)	69.1% (16.7–100%)	44.2% (range 0-100%)	46.0% (range 16.7–100%)
Average percentage of participants within a cluster who reported using the height-adjustable workstation > few times a week	N/A	95.28% (87.5–100%	N/A	95.12% (range 84.21–100%)	N/A	92.24% (range 75.0-100%)	N/A	68.98% (range 33.33–100%)

^a^ Clusters reaching the high level of implementation and engagement would also meet the medium level

^b^ Clusters reaching the high and medium level of implementation and engagement would also meet the low level

**Table 7:** Level of implementation and engagement determined by percentage of implementation and engagement (as outlined in Table 3)

	Level 1: High implementation and engagement		Level 2: ≥Medium^a^ implementation and engagement		Level 3: ≥Low^b^ implementation and engagement		Level 4: Limited or no implementation and engagement	
Intervention components	SWAL	SWAL + desk	SWAL	SWAL + desk	SWAL	SWAL + desk	SWAL	SWAL + desk
**Clusters** **Participants**	**8 (29.6%)** **80 (32.1%)**	**5 (20%)** **41 (17.1%)**	**19 (70.4%** **196 (78.7%)**	**12 (48%)** **113 (47.1%)**	**21 (77.8%)** **213 (85.5%)**	**22 (88%)** **221 (92.1%)**	**6 (22.2%)** **36 (14.5%)**	**3 (12%)** **19 (7.9%)**
No. of clusters who had a workplace champion trained and stayed in the role past the first 3 months	8 (100%)	5 (100%)	19 (100%)	12 (100%)	21 (100%)	22 (100%)	2 (33.3%)	2 (66.7%)
Average percentage of participants within the clusters who completed the online education	91.81% (range 77.78–100%)	94.44 (range 83.33–100%)	87.98% (range 66.67–100%)	88.94% (range 66.67–100%)	86.35% (60.00-100%)	80.88% (range 33.33–100%)	43.75% (range 0-100%)	71.1% (33.3–100%)
Average percentage of the monthly emails sent by workplace champions	81.0% (range 30.0-100%	88.4% (range 60.0-100%)	87.0% (range 30.0-100%)	83.3% (range 22.2–100%)	85.4% (range 30.0-100%)	76.4% (range 0-100%)	33.3% (range 0-100%)	73.3% (range 20.0-100%)
No. of clusters who delivered 1 out of 2 group catch ups	8 (100%)	5 (100%)	18 (94.7%)	11 (91.7%)	20 (95.2%)	19 (86.4%)	2 (33.3%)	2 (66.7%)
No. of clusters who delivered 2 out of 3 sitting less challenges	7 (87.5%)	5 (100%)	18 (94.7%)	10 (83.3%)	19 (90.5%)	17 (77.3%)	2 (33.3%)	1 (33.3%)
Average percentage of the cluster who used any self-monitoring tool	86.7% (range 75.5–100%)	85.1% (range 75.0-100%)	76.1% (range 50.0-100%	69.8% (range 50.0-100%)	73.0% (range 40.0-100%)	62.6% (range 25.0-100%)	12.9% (0-100%)	24.4% (16.7–40.0%)
Average percentage of participants within a cluster who reported using the height-adjustable workstation > few times a week	N/A	95.28% (87.5–100%	N/A	90.26% (62.5–100%)	N/A	81.22 (range 33.3–100%)	N/A	80.0% (range 60–100%)

^a^ Clusters reaching the high level of implementation and engagement would also meet the medium level

^b^ Clusters reaching the high and medium level of implementation and engagement would also meet the low level

**Fig. 2:**
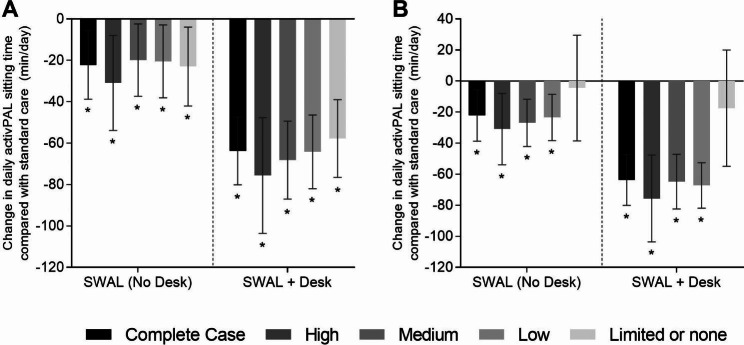
**a-b**. Changes (Adjusted mean difference and 95% CI) at follow-up) in daily sitting time (min/day) between participants randomised to standard care or intervention groups (SWAL only and SWAL plus desk) for the complete case analysis and by level of intervention implementation and cluster engagement. Figure **2a** shows data for the number of intervention components implemented and engaged with. Figure **2b** shows data for the percentage of implementation and engagement with each intervention component

Figure 2a and b show the changes in daily sitting time at 12 months for the whole sample and by level of intervention implementation and cluster engagement. In the complete case analysis, the SWAL only and SWAL plus desk intervention groups sat for 22.2 (95% CI -38.8 to -5.7, *p* = 0.003) and 63.7 (95% CI -80.1 to -47.4, *p* < 0.001) fewer minutes/day compared to the control group respectively at the 12 month follow up. Those clusters in the SWAL only and SWAL plus desk intervention groups who implemented and engaged with the most intervention components (Fig. 2a) and had the highest percentage (Fig. 2b) of cluster implementation and engagement sat for 30.9 (95% CI -53.9 to -7.9, *p* = 0.003) and 75.6 (95% CI -103.6 to -47.7, *p* < 0.001) fewer minutes/day compared to the control group respectively at 12 month follow up. The differences in sitting time were lower for the ≥ medium and ≥ low levels of implementation and engagement than the higher levels for both the number of intervention components (Fig. 2a) implemented and engaged with (SWAL ≥ low: -20.5 (-38.1 to -2.9), *p* = 0.002; SWAL ≥ medium: -19.8 (-37.3 to -2.4), *p* = 0.003; SWAL + desk ≥ low: -64.2 (-82.0 to -46.4), *p* < 0.001; SWAL + desk ≥ medium: -68.2 (-87.0 to -49.4), *p* < 0.001)) and the percentage (Fig. 2b) of implementation and engagement (SWAL ≥ low: -23.4 (-38.4 to -8.5), *p* = 0.002; SWAL ≥ medium: -27.0 (-42.2 to -11.7), *p* = 0.001; SWAL + desk ≥ low: -67.2 (-81.9 to -52.6), *p* < 0.001; SWAL + desk ≥ medium: -64.8 (-82.4 to -47.1), *p* < 0.001). The differences, however, were similar between ≥ medium and ≥ low levels. The differences in sitting time observed for the ≥ medium and ≥ low levels were similar to the complete case analysis (differences in sitting time within five minutes/day of the complete case analysis). When examining the number of intervention components (Fig. 2a), the differences in sitting time observed for the clusters with limited or no implementation and engagement were also similar (-22.97 (-42.03 to -3.91), *p* = 0.02) to those observed for the ≥ low and ≥ medium levels for the SWAL group and the complete case analysis but were slightly lower for the SWAL plus desk group (-57.77 (-76.55 to -38.98), *p* < 0.001). These differences in sitting time were significantly different from the control group. However, when examining the percentage (Fig. 2b) of implementation and engagement, the differences in sitting time for clusters in the limited or no category were lower and not significantly different from the control group (SWAL − 4.45 (-38.49 to 29.58), *p* = 0.80; SWAL + desk − 17.46 (-54.91 to 20.00), *p* = 0.35).

## Discussion

The SWAL intervention successfully reduced daily sitting time over a 12 month follow up and relative to controls, the provision of the height-adjustable workstation, alongside the SWAL intervention, produced greater intervention effects than the SWAL intervention on its own. The purpose of this paper was to describe the level of intervention implementation and engagement and explore whether changes in sitting time differed by levels of intervention implementation and engagement. This is an important issue to address in intervention research and allows for advances in understanding why and how, in addition to whether, behaviour change may or may not have occurred.Overall, it is clear from the process evaluation that the SWAL intervention was not delivered in its entirety, with variation across clusters and participants in the intervention strategies that were implemented and engaged with. Our exploratory analysis suggests that this variation impacts on intervention effectiveness, with greater changes in sitting time observed for those in both intervention groups who had high levels of implementation and engagement.

Implementation fidelity is the degree to which an intervention is delivered as intended [28]. The SWAL intervention was uniquely implemented in an ecologically valid ‘real world’ context where the research team had no control over the intervention delivery; this was the responsibility of the workplace champions. The workplace champions were volunteers, with the Senior Management team in the workplace approving two hours of protected time each month to perform the role. Our study was successful in recruiting and training employees within the councils to volunteer as workplace champions to facilitate the intervention. However, only 44.4% (SWAL only) and 36.0% (SWAL plus desk) of champions delivered 100% of the main intervention strategies. A similar intervention, where a train-the champion approach was used for intervention delivery, also reported implementation issues in the early adopters version of the programme (where participants received no external support for the uptake, implementation or evaluation of the programme) [29]. Healy and colleagues [29] reported that only 5.2% of champions implemented all seven core elements of the program and less than 20% reached the final review stage of the programme, with these findings used to optimise the programme and associated toolkit to ensure it was fit-for-purpose for a national implementation trial [30]. Healy [29] also reported that 36% of champions in the early adopters phase failed to complete any steps in the programme, whereas in SWAL this was zero in the SWAL plus desk group and 14.8% in the SWAL only group. However, although our intervention was facilitated by workplace champions, it was still part of a randomised controlled trial with researcher contact during the evaluation and therefore this is likely to have led to higher implementation.

The implementation issues with SWAL might be attributable to workplace champion and participant burden, such as organising the group catch-up sessions and sitting less challenges, setting up and using the self-monitoring tools, and operating the height-adjustable desk itself. Moreover, the retention of the workplace champions was a challenge. However, at the end of the study workplace champions rated the time burden of the role on a scale of 1–10 (1 = not at all time consuming to 10 extremely time consuming) and reported a mean score of 4.7. This suggests reasons other than the workload associated with facilitating the intervention for not implementing the intervention as intended and/or dropping out of the role. Furthermore, many participants mentioned that their day-to-day workload was a significant barrier to engagement. These challenges concerning intervention fidelity suggest that greater efforts are required in future interventions, either at the intervention development stage, or for the prompting and maintenance of intervention strategies. In addition, the use of workplace volunteers taking on these important roles may require further assistance, and more work is required in order to investigate how to optimally incorporate such volunteers, given that these volunteers within the organisation have been shown to be more effective than external interventionists at promoting behaviour change [14]. Further work could explore whether larger groups may benefit from having more than one champion to spread the workload of facilitating the intervention, developing interventions with a greater focus on BCTs that require low cognitive effort from employees such as restructuring the physical and social environment and therefore reduced time commitment from workplace champions and exploring how to integrate this type of intervention within an organisation’s wider occupational safety and health protection and health promotion approaches (i.e., a total worker health strategy) [31]. User centred designs, and use of strategies such as PPI (patient and public involvement), are important in this context [30]. Involvement of multiple stakeholders, as well as allowing for flexibility and tailoring to suit the workplace, require careful consideration. PPI was utilised for the present study, although further developments, such as co-production of the intervention with participants, are also possible.

Most of the intervention strategies in SWAL were delivered to some extent across the clusters but there was large variation. Engagement with online education session was high with 81% of participants completing it. This was similar attendance to a face-to-face education session in our previous intervention SMArT Work (86% of participants attended) [32]. Despite content of the monthly emails, group catch up sessions and sitting less challenges pre-defined for the workplace champions, the delivery of these was more varied across clusters and a proportion of participants did not engage with them at all. Furthermore, despite evidence indicating the importance of self-monitoring and prompts for behaviour change [27, 33] uptake for this aspect of the intervention was low compared to some of the other intervention strategies.

Our exploratory analysis showed that regardless of intervention arm, being in the highest group for implementation and engagement was associated with superior behaviour change effects. There was an approximate 10 min larger reduction in sitting time for Level 1 (high) compared to Level 3 (≥ low). Moreover, Levels 2 (≥ medium) and 3 (≥ low) appeared to have very similar effects to each other. It is instructive that the reductions in sitting achieved by the SWAL (no desk) arm at Level 1 of implementation and engagement, relative to controls, were much less than for the SWAL plus desk arm at Levels 2 and 3. This is suggestive of strong effects for environmental restructuring. Overall, superior effects were shown for those in the SWAL plus desk arm and with high levels of implementation and engagement, as expected.

For the number of intervention components implemented and engaged in (see Table 2), it is likely that the key differentiating factor between Level 1 and other levels is that of self-monitoring which, for the current study included some prompts to remind the participant that had been sitting or using their computer for too long without a break (used as a proxy for sitting). Given that Levels 2 and 3 were similar in terms of the change in sitting time, it is only self-monitoring (and specifically 75% or more of the cluster using some form of self-monitoring) that is unique to Level 1. Self-monitoring has been identified as an important behaviour change technique [33] under the label of ‘behavioural regulation’ [34] and has been identified as a particularly promising BCT for sedentary behaviour interventions [27]. This is supported by the present findings. Moreover, self-monitoring may play several roles for behaviour change, including goal-setting, feedback, and enhancement of self-efficacy. A meta-analysis by Compernolle and colleagues [35] showed that interventions using self-monitoring tools significantly reduced sedentary time in the short term, with sub-group analyses pointing to device-based tools, such as a Fitbit, Garmin or Jawbone, being key. For SWAL participants we suggested a range of freely available computer and mobile applications to monitor their sitting time and/or provide prompts to break up sitting, with many participants also choosing to purchase wearable technologies, such as a Fitbit. These types of tools have been shown to be effective in reducing sitting time up to 6 month follow up but not beyond this [36], although only three studies in this meta-analysis had a follow up longer than 6 months. The current study adds to the limited number of longer term studies and demonstrates the potential effects of self-monitoring and prompts over a 12 month period.

Future studies of this kind may wish to see how certain BCTs operate alone or when additional BCTs are added. For example, Schroé et al. [37] showed that different BCTs, and combinations of BCTs, may act differently for physical activity and sedentary behaviour in e- and m-health contexts.

For the percentage of implementation and engagement (see Table 3), enhanced behaviour change was observed for Level 1 (~ 8 min in comparison to the complete case analysis), however differences in sitting time were significantly different from the control group for Levels 1, 2 and 3 for both the SWAL and SWAL plus desk groups, with only small differences between the 3 levels (~ 7–8 min between level 1 and 3). Level 4, limited or no implementation and engagement, resulted in no significant differences in sitting time compared to the control group. This suggests that clusters need to implement at least 25% of each of the intervention strategies and have at least 25% of the cluster engage in them in order for a difference in sitting time to be achieved. Furthermore, this demonstrates that being in the study alone and being measured is not enough to lead to behaviour change.

Despite the ≥ low and ≥ medium levels being less effective, they still led to reductions in sitting time compared to the control group. Furthermore, significant differences in sitting time were still found for those clusters with more limited implementation and engagement (level 4) when examining the number of intervention components (but keeping engagement with the included component high).Additionally, differences in sitting time for all levels were similar to or exceeded those seen in the complete case analysis. It is clear from these data that many of the clusters and participants in level 4 still implemented and engaged with various aspects of the intervention (as shown in Table 5). This suggests that a more pragmatic and less burdensome intervention may still be beneficial, and cost effective based on the behaviour change observed [19], for those workplaces/offices who are unable deliver and/or engage with all intervention components. For example, provision of a height-adjustable workstation with education may still yield meaningful behaviour change even without other strategies offered in the present intervention. Small scale before and after studies have shown that this type of intervention can reduce sitting time in the short term (one to three months) [38]. Future studies should aim to address what the minimal intervention might be and designs, such as the multiphase optimisation strategy (MOST) [39, 40] need consideration in this regard. In the context of the current study, MOST proposes that before further trials are undertaken (to build on SWAL), components need to be identified that are active in an intervention and, then, which doses of each component lead to the best behaviour change outcomes [40]. The current secondary data analysis makes some progress in this regard, but further work utilising MOST is required to address these questions. Moreover, it might be that the active ingredients vary by organisation and worker characteristics; thus, a menu of known effective intervention strategies may be more appropriate.

It was clear from the percentage of cluster implementation and engagement data that sitting time was not different from the control group when there was very little or no intervention implementation or engagement, as demonstrated in Table 6. A key difference here appeared to be the low number of clusters that still had a workplace champion after 3 months and a much lower percentage of implementation and engagement across all intervention components in comparison to the ≥ low, ≥medium and high levels, with the exception of desk use which remained high at 80%.

For the testing of intervention implementation and engagement, the study has both strengths and limitations. The study is one of very few that has addressed the issue of sedentary behaviour implementation and participant engagement. This allows for progress to be made in better understanding how and why an intervention might work and takes us beyond the typical answer of whether it worked. Moreover, we had a range of important implementation and engagement assessments at individual and cluster level. Limitations of the study include data not being available for all participants and all clusters therefore an under- or over-estimation of implementation and engagement was possible. Additionally, this is a secondary unplanned analysis. The study was not powered to assess change in sitting time by intervention implementation and engagement level and the multiple testing may have increased the chance of a type one error. The study recruited participants from local government only, therefore results may not be generalisable to other employment sectors. Furthermore, we only included participants in the current analysis if they had at least one valid day of activPAL data, this could have been any day of the week. However, our previously published sensitivity analysis including only participants who provided at least 4 valid days of data showed similar results to the complete case analysis using 1 valid day of data [15], indicating robustness of the results, Finally, the analysis approach breaks randomisation by excluding clusters/participants based on their intervention implementation and engagement level – we acknowledge that this may overestimate effect sizes.

## Conclusions

In summary, the SWAL intervention was not delivered in its entirety in a large amount of clusters in both intervention groups. Most of the intervention strategies were delivered to some extent across the clusters although there was large variation. Our exploration of the effects of measures of intervention implementation and engagement showed superior effects for the reduction of daily sitting time for those in the SWAL and SWAL plus desk intervention arms who had high levels of implementation and engagement. Lower levels of implementation and engagement were associated with ~ 10 min/day lower reduction in sitting time compared to high levels but differences in sitting time were still significant compared to the control group. Overall, reductions in daily sitting time were most evident for those with a height adjustable workstation, but the highest level of implementation and engagement was also helpful. Future research could use research designs to understand the minimal intervention needed to elicit meaningful behaviour change.

### Electronic supplementary material

Below is the link to the electronic supplementary material.

Supplementary Material 1

## Data Availability

The datasets used and/or analysed during the current study are available from the corresponding author on reasonable request.
