# Supplementary material for: Implementation and engagement of the SMART Work & Life sitting reduction intervention: an exploratory analysis on intervention effectiveness
Source: Int J Behav Nutr Phys Act. 2023 Dec 19;20:148. doi: 10.1186/s12966-023-01548-5 (PMC10729557; doi:10.1186/s12966-023-01548-5)
Supplement: Supplementary file 1 — Supplementary Material 1 [file 12966_2023_1548_MOESM1_ESM.docx]

**Response to reviewers**

| **Comments** | **Response** |
| --- | --- |
| **Editor comments** |  |
| The role of funding body needs to be included as per journal guidelines. Please note that the section under the heading “Declarations” must be included in your manuscript and must include the subheadings in the order listed below. Under the subheading “Funding”, you must declare the role of the funding body in the design of the study and collection, analysis, and interpretation of data and in writing the manuscript. | The declarations were included in the original manuscript and in the correct order. However, we have now added the statement about the role of the funding body which was missing from the original subheading on funding.  ‘*The funder had no role in the design of the study, the collection, analyses or interpretation of data, or the writing of the manuscript*.’ |
| **Reviewer 1 Comments** |  |
| Abstract:  Please insert "model or analyses" in the sentence starting by Mixed effects at line 54, after regression. | We have added the word ‘analyses’. |
| Abstract:  Please rephrase the sentence starting Across in line 60 to give meaning. | We have amended the sentence slightly:  ‘*Across remaining clusters, the level of intervention implementation varied*.’ |
| Abstract:   Please re-consider the relevance of the sentence starting these in line 67. | We have considered this suggestion but this sentence puts the results into context of the main analyses for the study (published in BMJ) so we have decided not to change the text. |
| Abstract:   Please include what the levels mentioned in line 69 refers to. | We are unsure what the reviewer is referring to here as the text mentions low and medium levels.  ‘*The differences in sitting time observed for the medium and low levels were similar to the complete case analysis*.’ |
| Background:  The background section is quite long and elaborate, please consider to move some sections to the methods - without repetition of information. For instance the section from line 122-133 could be considered to be moved to the methods section. | We agree that there was some repetition in the information provided in the introduction and methods. Therefore, we have deleted some of the suggested text around the SMART Work & Life intervention (see pages6-7, lines 122-149) and expanded briefly on the text in the methods describing the intervention (page 10, lines 211-213). |
| Background:  The background should also be supplied with argumentation of why standing is preferable over sitting in office work, which may not be the case from a physiologic perspective as standing instead of sitting may not solve any health issues, but rather introduce a shift in which health issues becomes most prevalent. | We feel that this does not fit into the introduction as we are not justifying why the intervention is needed and its focus (information on this is provided in the protocol paper describing the study and the main quantitative results paper published in BMJ). The focus of the current paper is the need to understand to what extent the intervention was implemented and engaged with. Furthermore, the intervention was not focused on encouraging prolonged standing, which as the reviewer highlights can also lead to health problems. The participants were encouraged to reduce their sitting time with standing and moving around and regularly break up sitting time every 30 minutes for a few minutes (with standing and/or moving). It was emphasised to participants, particularly in the SWAL+Desk group, that regular posture changes were key (from sitting to standing and standing to sitting) whilst using their height-adjustable desk. Posture changes were encouraged every 30 minutes. Our intervention did show reductions in sitting, most of which were replaced by standing but this was standing time incorporated across the work day in bouts, not in one long chunk. |
| Background:  Please include whom were providing the support, mentioned in line 136. | We have now included the following information on who provided the training and support in the methods section (page 10, lines 222-227):  ‘*workplace champions (one or two per cluster depending on the size of cluster) were trained to facilitate the intervention and were provided with training and support (from a team within the Leicester Diabetes Centre (independent of the core research team) experienced in providing training for delivering structured education programmes).*’ |
| Background:  Please include how the workplace champions were selected, in line 137. | This information is included in the methods section and we feel it is better suited in the methods (page 9, lines 192-195).  ‘*To be eligible, each cluster was required to have at least one participant who was willing to act as workplace champion. Participants were asked to indicate on the information form if they were willing to take on this role*.’ |
| Methods: Please include a description/cut-point (ex XX% of an weekly working time of XX hours) of the criteria of the majority of the day, mentioned in line 178 and the equivalent number of hours corresponding to the 60% of full time, mentioned in line 179. Specificity in the number of hours increase the comparability of the population to other non-British working populations. | We have added ‘≥50%’ to the text. It is more difficult to add hours against 60% of full time as some employees were on different contracts i.e., full time hours for more senior employees may have been 37.5 hours but for some employees this may have been 35 hours. We have however, provided an example for someone on a 35 hour per week contract (page 9, lines186-187):  ‘*worked at least 60% full-time equivalent (e.g., ≥21 hours for those on a 35 hour working week contract)*’. |
| Methods: Please specify whether the workplace champion were selected or volunteered, line 185 | This information was included in the sentences that followed line 185 (page 9, now line 193):  ‘*Participants were asked to indicate on the information form if they were willing to take on this role’* |
| Methods: Please specify with whom and in which setting the face-to-face training took place, line 216 | Information on who delivered the training has been provided in response to an earlier comment (page 10, lines 222-227). We have also added where the training took place (page 12, lines 238-239):  ‘*Each cluster was required to have an individual attend face-to-face training (took place at their workplace) to become a workplace champion for the intervention’*. |
| Methods: Please only mention the aim once, thus consider to delete the mentioned aim in line 230-232 | We have decided to leave the aims in as they help to put the methods described to define levels into context of each aim. |
| Methods: Please add what the three levels are off in line 232 | The three levels are defined on page 13, lines 255 - 271 as follow:  ‘*The three levels presented in Table 2 were defined by the study team after database lock, but prior to this analysis. To meet the criteria for the top level (Level 1) of intervention implementation and engagement, a cluster were expected to have implemented and engaged with all of the planned main intervention strategies. Specifically, they must have had a workplace champion trained and for them to remain in the role for ≥3 months, the champion must have delivered a high percentage of the ongoing support strategies (i.e., monthly emails, group catch up session and sitting less challenges), and a high percentage of the cluster (≥75%) must have engaged with the individual strategies (online education, self-monitoring, and height-adjustable desk use (SWAL plus Desk group only)), with the level of engagement required specified in Table 2. The medium level (Level 2) was the same as the top level but the requirement to engage with the self-monitoring strategy was removed. The low level (Level 3) was the same as the medium level but the requirement to implement the ongoing support strategies (emails, group catch up and challenges) was removed*.’ |
| Methods: The inclusion criteria of one valid day, but no requirement of this one day to be a work or non-workday, line 287, may hold some limitations (daily physical actiivity patterns are expected to vary across work and non-workdays and this may impact the results as you don't know the distribution of work and non-workdays) - thus, please include this selection of valid days in the limitations of the study | We have now added the following text to the limitations section in the discussion (page 30, lines 607-613):  ‘*Furthermore, we included participants in the current analysis if they had at least one valid day of activPAL data, this could have been any day of the week. However, our previously published sensitivity analysis including only participants who provided at least 4 valid days of data showed similar results to the complete case analysis using 1 valid day of data (13), indicating robustness of the results*.’ |
| Methods: Please add the definition of a complete case, mentioned in line 299 | We have now added the following text (page 16, lines 322-323):  ‘*was analysed on a complete case basis (i.e., only participants with valid activPAL data were included) using a linear multilevel model*.’ |
| Methods: Please specify in the statistical analysis section whether or not (and why) the analysis included nesting of subjects in companies/clusters. If you do not nest the subjects in the companies/clusters this should be mentioned in the limitations as the analysis thereby assume all participants from the same companies/clusters being alike - which you argue they are not. | The text states that office clusters were included as a random effect, therefore the analysis did nest participants within clusters (page 16, line 329):  ‘*Office clusters were included as a random effect*.’ |
| Results: Please add the distribution of the clusters with missing champions across the intervention arms, ex line 320 | We have now added additional text to show which intervention arms the champions were from (page 17, lines 342-346):  ‘*Workplace champion training attendance data were available for all clusters, with 51/52 clusters having at least one champion trained (one cluster from SWAL only did not have a champion trained). Within the first three months of the study, four champions (two from SWAL only and two from SWAL+Desk) withdrew from the role with no replacement being found (9.6% of clusters without a champion).*’ |
| Results: Please add to the discussion whether or not the amount of clusters implementing all of the main intervention strategies are expected or not - compared to other studies, mentioned in line 332 | In our original manuscript we had included a discussion of how our level of implementation and engagement compares to a previous similar study. This was included on page 21-22, lines 21-25 on page 21 and lines 1-9 on page 22 (now pages 23-24, lines 455-470):  ‘*However, only 44.4% (SWAL only) and 36.0% (SWAL plus Desk) of champions delivered 100% of the main intervention strategies. A similar intervention, where a train-the champion approach was used for intervention delivery, also reported implementation issues in the early adopters version of the programme (where participants received no external support for the* *uptake, implementation or evaluation of the programme) (27). Healy and colleagues (27) reported that only 5.2% of champions implemented all seven core elements of the program and less than 20% reached the final review stage of the programme, with these findings used to optimise the programme and associated toolkit to ensure it was fit-for-purpose for a national implementation trial (28). Healy (27) also reported that 36% of champions in the early adopters phase failed to complete any steps in the programme, whereas in SMART Work and Life this was zero in the SWAL plus Desk group and 14.8% in the SWAL only group. However, although our intervention was facilitated by workplace champions, it was still part of a randomised controlled trial with researcher contact during the evaluation and therefore this is likely to have led to higher implementation*.’  Other large scale workplace interventions focused on reducing sitting have not reported fidelity or engagement data so we are unable to expand on this text. |
| Results: A ) and a space are missing in line 356 | Thank you for highlighting this. It has now been amended. |
| Discussion:  You mention greater efforts are required in line 21 page 22, but could other efforts, not necessarily greater, also have an impact? Ex more structural/systemic approaches like changing all tables in the conference rooms to standing height, highlighting standing as a strategic goal of the company health policy | As described in the methods section the SMART Work and Life intervention consisted of organization, environmental, group and individual behavior change strategies. Within the environmental strategies, the managers, champions and participants were encouraged to look at their working environment and make small scale restructuring changes for example adding high tables to meeting areas or eating areas (if this was something they felt would work for their group and be feasible). The groups were also encouraged to look at any policy changes they could make. However, as described in the introduction some BCTs within SMART Work and Life had an element of choice of strategies within the four strategies above (e.g., what small environmental changes to make) so implementation of environmental changes could vary across clusters and all, none or some may have chosen to make changes such as including more standing height tables. This specific detail for each cluster was not captured.  We have added some additional text in the discussion to suggest that future research could have a greater focus on the environmental changes suggested by the reviewer (page 25, lines 490-492):  ‘*Further work could explore whether larger groups may benefit from having more than one champion to spread the workload of facilitating the intervention or developing interventions with a greater focus on BCTs that require low cognitive effort from employees such as restructuring the physical and social environment and therefore reduced time commitment from workplace champions*.’ |
| Discussion: Could a group of champions at each cluster be considered to share the workload? | This is a good point and in our study where we had more than one person volunteer as workplace champion for the cluster, they could either share the role or decide amongst themselves who would take this on. Of the 51 clusters that had champions, we only had 5 which had more than 1 champion, which prevents us from understanding whether this had an impact on intervention fidelity and engagement. We have however, added in some text in the discussion for future research to consider this (page 25, lines 488-490):  ‘*Further work could explore whether larger groups may benefit from having more than one champion to spread the workload of facilitating the intervention*.’ |
| Discussion:  Further developments, mentioned in line 6, page 23, could also include considerations on using integrated approaches like the total worker health or work health improvement network concepts | Thank you for highlighting this. We have now made reference to this in the discussion (page 25, lines 493-495):  ‘*Further work could explore whether larger groups may benefit from having more than one champion to spread the workload of facilitating the intervention, developing interventions with a greater focus on BCTs that require low cognitive effort from employees such as restructuring the physical and social environment and therefore reduced time commitment from workplace champions and exploring how to integrate this type of intervention within an organisation’s wider occupational safety and health protection and health promotion approaches (i.e., a total worker health strategy).*’ |
| Discussion: The paragraph stating at line 19, page 23, should also include a discussion of program/delivery failure - as these results seems to be due to a delivery failure | The extent of the intervention delivery (what has and has not been delivered/engagement with) is highlighted and discussed on pages 23-24 and compared to other ‘real world’ implementation workplace interventions targeting sitting. We have chosen to discuss intervention delivery through the different levels of implementation and engagement which seems to be the converse of discussing ‘failure’ to implement. Therefore we have not added additional text. |
| Discussion: The mentioning of self-monitoring to transiently decrease the sedentary time, line 1, page 25, and that this is the only difference from other levels to level 1 - how would you expect the long term >6 months intervention effects to look? Please include this notion in the discussion of the generalizability and prospective effects from the study. | We are unclear what the reviewer is asking here as the current study demonstrates the longer (>6 months) term intervention effects as the follow up data presented is for 12 month follow up. We have however amended the text to clarify this as follows in the discussion (page 27, lines 542-546):  ‘*These types of tools have been shown to be effective in reducing sitting time up to 6 month follow up but not beyond this (35), although only three studies in this meta-analysis had a follow up longer than 6 months. The current study adds to the limited number of longer term studies and demonstrates the potential effects of self-monitoring and prompts over a 12 month period*.’ |
| Discussion: Please include how this mentioning of behavior change from provision of height adjustable tables only have worked out for other studies, line 5, page 26 | This is difficult to compare given there are only a handful of studies that have used a height-adjustable desk alone and these studies have been small scale (e.g., ~20-50 participants) with short term follow up (e.g, 1-3 months) and not cluster randomized controlled designs. We have however, added a brief mention of the review conducted in 2018 on workplace interventions to reduce sitting time (page 28, lines 573-580):  ‘*For example, provision of a height-adjustable workstation with education may still yield meaningful behaviour change even without other strategies offered in the present intervention. Small scale before and after studies have shown that this type of intervention can reduce sitting time in the short term (one to three months) (37). Future studies should aim to address what the minimal intervention might be and designs, such as the multiphase optimisation strategy (MOST) (38,39) need consideration in this regard*.’ |
| Discussion: Elaborate by explaining what further work should include, line 12, page 26 | The statement on further work was referring to the aforementioned text regarding conducting studies utilising MOST. We have amended the text slightly to make this clearer (page 29, lines 584-585):  ‘*The current secondary data analysis makes some progress in this regard, but further work utilising MOST is required to address these questions*.’ |
| Discussion: Consider to include a discussion of what impact the 8 min difference in sitting time might have on health to argue for this intervention to be cost-effective, is it worth it to make all the efforts for gaining the additional 8 min reduced sitting time? | The cost-effectiveness of the intervention has been published (this has now been referred to in the methods section) and both the interventions (SWAL only and SWAL+Desk) were considered to be cost-effective from a public health perspective. This was based on the complete case values that are presented in the current paper as a comparison to the results of the four levels of implementation and engagement. While the difference in change in sitting time between the levels of implementation and engagement appears to be small, the low, medium and high levels were all associated with a behavior change similar or exceeding that seen in the complete case analysis, therefore indicating that all three levels of implementation and engagement could be cost-effective. We have now made reference to this in the discussion (page 28, lines 566-573):  ‘*Additionally, differences in sitting time for all levels were similar to or exceeded those seen in the complete case analysis. It is clear from the data that many of the clusters and participants in level 4 still implemented and engaged with various aspects of the intervention (as shown in Table 5). This suggests that a more pragmatic and less burdensome intervention may still be beneficial, and cost effective based on the behaviour change observed (18), for those workplaces/offices who are unable deliver and/or engage with all intervention components*.’ |
| Discussion: Please include whether these results are generalizable and to whom, line 12, page 27 | We have now added the following text to acknowledge that participants were only recruited from local government (page 30, lines 607-609):  ‘*The study recruited participants from local government only, therefore results may not be generalisable to other employment sectors*.’ |
| **Reviewer 2 Comments** |  |
| i) A flow chart showing the areas, clusters, participants, and workplace champions would be helpful for the reader. The chart would benefit from showing the participant-flow from the six local Councils. | A new figure has now been included which shows the number of clusters, participants and workplace champions from each area in the UK and council (see Figure 1). |
| ii) The intervention activities are now included in additional file 1. In my opinion, it would be easier for the reader if the information was presented in a table integrated into the text. If the current table based on the time points seems too large, a compressed table based on each activity could be an alternative. | As suggested we have summarized the workplace champion schedule in a table in the main text (see Table 1). |
| iii) How was the sitting time estimated from the accelerometer data? That is, what is considered sitting time? This information is undoubtedly presented in the main article but in my opinion, it should be mentioned also here. | There was a paragraph in the original manuscript that detailed how sitting time was assessed (originally page 13, lines 272-288 – now page 15, lines 296-311):  ‘*The activPAL3™ micro accelerometer-based device (PAL Technologies Ltd, Glasgow, Scotland, UK) was used to assess average daily (i.e., all waking hours) sitting time. Participants were asked to wear the device 24 hours a day for 8 days whilst also completing a sleep diary which recorded the times they got into bed, went to sleep, woke up and got out of bed. The activPAL was initialised with default settings and the device was waterproofed with a nitrile sleeve and attached to the midline anterior aspect of the thigh using a transparent dressing. On device return, data were downloaded using PAL Connect and event files were exported. Event files were then uploaded into the freely available Processing PAL java application (University of Leicester, Leicester, UK available at: https://github.com/UOL-COLS/ProcessingPAL) to be cleaned and processed as described in Edwardson et al (13). Once data were cleaned, average daily sitting time was calculated from any valid days. A valid day was defined as having ≥10 hours wear time per day, ≥1,000 steps per day, and <95% of the day spent in any one behaviour. To be included in the analysis participants were required to have at least one valid day (could be any day of the week).*’ |
| iv) Page 19, lines 11-20: Too long sentence - please revise. | Thank you for highlighting this. The sentence appears long because of the values given in brackets. However, we have shortened it slightly but creating a new sentence with the final information:  ‘*The differences, however, were similar between ≥medium and ≥low levels*.’ |
| v) Figure 1a-b: Please indicate to the reader what the abbreviation ITT means? Preferably you should also mention ITT in the statistical analysis instead of using "... analysed on a complete case basis..." | Thank you for highlighting this, it was an error and should have been labelled ‘Complete case’. This has been amended on the figure. |
